# Constant Light Exposure Alters Gut Microbiota and Promotes the Progression of Steatohepatitis in High Fat Diet Rats

**DOI:** 10.3389/fmicb.2020.01975

**Published:** 2020-08-21

**Authors:** Lin Wei, Fangzhi Yue, Lin Xing, Shanyu Wu, Ying Shi, Jinchen Li, Xingwei Xiang, Sin Man Lam, Guanghou Shui, Ryan Russell, Dongmei Zhang

**Affiliations:** ^1^Department of Endocrinology, Xiangya Hospital, Central South University, Changsha, China; ^2^Department of Geriatrics, National Clinical Research Center for Geriatric Disorders, Xiangya Hospital, Central South University, Changsha, China; ^3^State Key Laboratory of Molecular Developmental Biology, Institute of Genetics and Developmental Biology, Chinese Academy of Sciences, Beijing, China; ^4^Cardiomatabolic Exercise Lab Director, Department of Health and Human Performance, College of Health Professions, University of Texas Rio Grande Valley, Brownsville, TX, United States

**Keywords:** non-alcoholic fatty liver disease, light pollution, gut microbiota, short chain fatty acids, gut-liver axis

## Abstract

**Background:**

Non-alcoholic fatty liver disease (NAFLD) poses a significant health concern worldwide. With the progression of urbanization, light pollution may be a previously unrecognized risk factor for NAFLD/NASH development. However, the role of light pollution on NAFLD is insufficiently understood, and the underlying mechanism remains unclear. Interestingly, recent studies indicate the gut microbiota affects NAFLD/NASH development. Therefore, the present study explored effects of constant light exposure on NAFLD and its related microbiotic mechanisms.

**Materials and Methods:**

Twenty-eight SD male rats were divided into four groups (*n* = 7 each): rats fed a normal chow diet, and exposed to standard light-dark cycle (ND-LD); rats fed a normal chow diet, and exposed to constant light (ND-LL); rats fed a high fat diet, and exposed to standard light-dark cycle (HFD-LD); and rats on a high fat diet, and exposed to constant light (HFD-LL). Body weight, hepatic pathophysiology, gut microbiota, and short/medium chain fatty acids in colon contents, serum lipopolysaccharide (LPS), and liver LPS-binding protein (LBP) mRNA expression were documented post intervention and compared among groups.

**Result:**

In normal chow fed groups, rats exposed to constant light displayed glucose abnormalities and dyslipidemia. In HFD-fed rats, constant light exposure exacerbated glucose abnormalities, insulin resistance, inflammation, and liver steatohepatitis. Constant light exposure altered composition of gut microbiota in both normal chow and HFD fed rats. Compared with HFD-LD group, HFD-LL rats displayed less *Butyricicoccus, Clostridium*, and *Turicibacter*, butyrate levels in colon contents, decreased colon expression of occludin-1 and zonula occluden−1 (ZO-1), and increased serum LPS and liver LBP mRNA expression.

**Conclusion:**

Constant light exposure impacts gut microbiota and its metabolic products, impairs gut barrier function and gut-liver axis, promotes NAFLD/NASH progression in HFD rats.

## Graphical Abstract

Constant light exposure alters microbiota and promotes NASH.

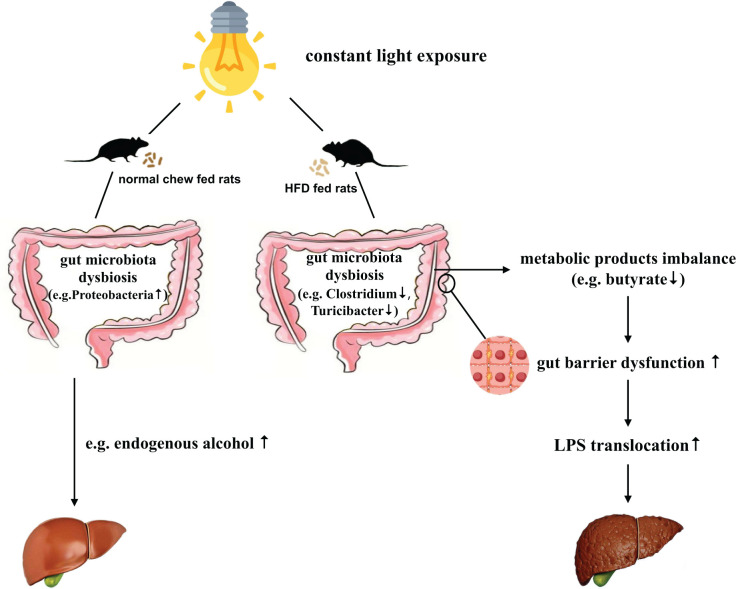

## Highlights

- Constant light exposure promotes NAFLD/NASH progression in HFD rats.
- Constant light exposure alters composition of gut microbiota.
- Constant light exposure impairs gut barrier function and gut-liver axis in HFD rats.

## Introduction

Non-alcoholic fatty liver disease (NAFLD) is defined as the presence of hepatic steatosis in at least 5% of hepatocytes which is not attributed to alcohol consumption or other secondary causes of steatosis (Cairns and Peters, 1983). Although steatosis has long been considered as a benign liver disease, it may progress into a more aggressive form of non-alcoholic steatohepatitis (NASH), which in turn may lead to cirrhosis and, sometimes, to hepatocellular carcinoma (Xanthakos et al., 2006). NAFLD is becoming the most common chronic liver disease worldwide, affecting about 20–30% of the general population (Sheka et al., 2020).

Although fatty liver and steatohepatitis most commonly stem from overnutrition and lack of exercise, additional mediators, such as environmental factors, have recently been postulated (Eslam et al., 2018). One novel environmental risk factor for NAFLD is light pollution. Light pollution, defined as the alteration of natural light levels due to the introduction of artificial light at night, is a major side-effect of urbanization (Falchi et al., 2016). Artificial light allows people to extend daytime activities into the night and engage in countercyclical nighttime shift work. As such, it may adversely affect health via circadian rhythm disruption (Lunn et al., 2017).

Circadian rhythms refer to physiological processes that occur with a repeating period of approximately 24 h, and ensure that internal physiology is synchronized with the external environment (Gachon et al., 2004). In mammals, suprachiasmatic nuclei (SCN) acts as the master circadian clock. SCN communicates time-of-day information by synaptic and diffusible signals to clocks in various brain regions and peripheral organs (i.e., peripheral clock). Light information is transmitted by way of the retino-hypothalamic tract connecting the eye to the SCN and is the most potent synchronizing factor and the main zeitgeber that synchronizes the clock with the external environment (Panda et al., 2002). Thus, the SCN serves to synchronize the timing of rhythmic activities throughout the body to the light/dark cycle, and responds to light more rapidly than peripheral tissues (Welsh et al., 2010). Taken together, the endogenous biological clocks can be disrupted by nighttime light exposure (light pollution).

It is reasonable to suspect light pollution affects physiological function due to the importance of the circadian system in regulating homeostatic functions. Data show that circadian disruptions from light pollution cause significant disruptions in physiological function. Epidemiological evidence from shift workers exposed to high levels of light at night suggest that prolonged exposure to light at night increases the risk for cancer, mood disorders, and metabolic dysfunction (Kubo et al., 2011; Xiao et al., 2019). Additionally, the increase in exposure to light at night parallels the global increase in the prevalence of obesity and metabolic disorders (Koo et al., 2016), well-known risk factors for NAFLD. However, the effects of environmental light pollution on the development of NAFLD remains unclear.

The gut microbiota is recognized as an “external” organ playing an important role in host physiology and metabolism (Lozupone et al., 2012; Raman et al., 2013). Both animal and observational studies in NAFLD patients suggest links between gut microbiota changes and NAFLD (Chiu et al., 2017; Tripathi et al., 2018; Zhou et al., 2018). The changes of gut microbiota may disrupt the gut tight junctions, leading to increased gut permeability and LPS translocation. Increased LPS translocation induces “metabolic endotoxemia,” which triggers inflammatory reactions, insulin resistance, and promotes the development of NAFLD (Leung et al., 2016; Aron-Wisnewsky et al., 2020).

It has been reported that circadian disruption by constant light exposure changes gut microbiome taxa and their functional gene composition (Wu et al., 2018). However, data on the effects of light pollution on gut microbiome in NAFLD subjects is limited. High fat diet (HFD) feeding is an extensively used model of NAFLD in rodents. Accumulating evidence shows that HFD reduces microbial diversity and alters gut microbiota composition (Cani et al., 2008; Zhang et al., 2012). In the present animal study, we observed the effects of constant light exposure on NAFLD and explored changes of gut microbiota in the colon content in HFD-fed rats model of NAFLD.

## Materials and Methods

### Animal Experiment

Twenty-eight male Sprague-Dawley (SD) rats (6 weeks old) were purchased from Hunan Slac-Jingda Laboratory Animal Co. (Changsha, China). All rats were housed under specific pathogen-free conditions in a temperature-controlled room with free access to water and food. All rats were fed with normal chow diet (ND, fat 12%, carbohydrate 66%, protein 22%, 3.50 kcal/g) and under 12:12 h light/dark cycle for 1 week to adapt to the environment. The rats were then randomly divided into 4 experimental groups and placed in two separate rooms (*n* = 7 each): (1) ND-LD group: rats on a normal diet (ND), and exposed to standard light/dark (LD) cycle; (2) ND-LL group: rats on a ND, and exposed to constant light (LL); (3) HFD-LD group: rats fed on a HFD (fat 37%, protein 17.5%, carbohydrate 45.5%, 4.50 kcal/g), and exposed to standard LD cycle; and (4) HFD-LL group: rats on HFD, and exposed to constant light (LL). The LD rats were placed in a room with a standard 12 h light/dark (LD) cycle: lights on between 8 a.m. and 8 p.m., with the light intensity in the cage set at 200 lux. The LL rats were placed in a constant-light room where they were exposed to 200 lux of continuous light at cage level. The light sources were natural white fluorescent light tubes with a wavelength range of 400∼560 nm.

Food intake and body weight were recorded weekly for all animals throughout the experiment for 16 weeks. Animal protocols were approved by the Animal Use and Care Committee of Central South University and were conducted according to the regulations set by Central South University (No.2018sydw184).

### Intraperitoneal Glucose Tolerance Test (IPGTT) and Insulin Tolerance Test (ITT)

An IPGTT was performed in the 14th week of experiment after an overnight fast. The rats were given an intraperitoneal injection of 50% D-glucose (2.0 g/kg) after fasting. Blood samples were measured from the tip of the tail at 0, 15, 30, 60, and 120 min after glucose injection using a portable glucose monitor (ACCU-CHEK; Roche Diagnostics, Mannheim, Germany).

ITT was carried out 5 days after IPGTT. Insulin (0.75 IU/kg, Novolin R, Novo Nordisk, Denmark) was injected intraperitoneally after an overnight fast, and blood glucose measurement procedure was the same as IPGTT.

### Body Composition Assessment

Dual Energy X-ray Absorptiometry (DXA, GE Lunar Corp., United States) was utilized to assess body fat mass using small animal software (GE Medical Systems Lunar, Madison, WI, United States).

### Sample Collection

After 16 weeks of intervention, rats were sacrificed in 2 consecutive days from 8 a.m. to 12 p.m. after fasting for 12 h. They were sacrificed while under anesthesia (inhaled) with 1.5–3.0% isoflurane (RWD Life Since Co., China). The abdominal cavity was then rapidly opened, and blood samples were collected from the superior vena cava and centrifuged at 2000 × g for 20 min to isolate serum (stored at −20°C).

Liver tissues were collected and weighed. Liver tissues were then either fixed with 4% paraformaldehyde solution or frozen in liquid nitrogen and stored at −80°C until analysis.

Colon content samples were collected under a sterile fume to prevent miscellaneous bacterium contamination and then frozen in liquid nitrogen and stored at −80°C until they were analyzed for gut microbiota or short/medium chain fatty acids measurements.

Proximal colon segments were collected and then fixed with 4% paraformaldehyde solution for immunohistochemical analysis.

### Relative Visceral Fat Weight

Epididymal fat were quickly excised and weighed. The epididymal fat relative body weight was represented for relative visceral fat weight.

### Serum Biochemical Analysis

Serum levels of triglycerides (TG), total cholesterol (TC), low-density lipoprotein cholesterol (LDL-C), high-density lipoprotein cholesterol (HDL-C), alanine aminotransferase (ALT), and aspartate aminotransferase (AST) were measured using commercial reagents (Serotec Co., Sapporo, Japan) according to the manufacturer’s recommendations.

### Liver Pathology

Paraffin-embedded liver sections (5 μm) were stained by hematoxylin and eosin (H&E). The NAFLD activity score (NAS) was assessed by an experienced physiologist using indices of inflammation, steatosis, and hepatocyte ballooning as previously published (Kleiner et al., 2005).

### Gut Microbiota Analysis

Microbial DNA was extracted by FastDNA^TM^ Spin Kit for Soil (MP bio, United States) and quantified using NanoDrop 2000. The V3–V4 region of the 16S rRNA gene was intensified by PCR (94°C for 2 min, followed by 25 cycles at 94°C for 30 s, 55°C for 30 s, and 72°C for 1 min and a final extension at 72°C for 10 min) using primers 5′- CCTACGGGNGGCWGCAG -3′ for 341F and 5′-GACTACHVGGGTATCTAATCC -3′ for 805R. The Agencourt AMPure XP PCR Purification Beads (Bechman Coulter, United States) was used to purify the amplicons. The solution was checked for integrity using Agilent 2100 Bioanalyzer (Agilent Technologies, United States) and quantified using Invitrogen Qubit3.0 spectrophotometer (Thermo Fisher Scientific, United States). The Miseq Reagnent Kit V3 was used for normalization and sequencing on an Illumina MiSeq (Illumina, United States).

Adapter sequences and low-quality ends were removed using Trimmomatic v0.33. Fastq files were demultiplexed and quality-filtered with FLASH2. The operational taxonomic units (OTUs) which reached a 97% sequence identity were subjected to alpha-diversity analyses to evaluate samples biodiversity using mothur software (version 1.30.1). The R package (R 3.6.0) was used for the visualization of bacterial community classification and distribution. Linear discriminant analysis (LDA) effect size (LEfSe) was performed to identify taxonomic biomarkers that characterize the differences between groups with the logarithmic LDA score threshold set at 2.5. A generalized estimated equation (GEE) analysis was performed using R package to investigate whether changes of microbiota were associated with NAS. Functional capacity for each sequence was analyzed with phylogenetic investigation of communities by reconstruction of unobserved states (PICRUSt). The Kyoto Encyclopedia of Genes and Genomes (KEGG) analysis was used to identify credible biological functions.

### Short and Medium Chain Fatty Acids Measurement

Short chain fatty acids (SCFAs) and medium chain fatty acids (MCFAs) in colon contents were measured using HPLC-MS/MS method as previously described (Li et al., 2019). Briefly, samples were extracted with solvent mixtures containing acetonitrile and double distilled water and analyzed on a Thermo Fisher DGLC-3000 coupled to Sciex QTRAP 6500 Plus system. Octanoic acid-1-^13^C_1_ was used as an internal standard.

### Serum IL-6 and TNF-α

Serum IL-6 and TNF-α were measured using respective commercial rat-specific enzyme-linked immunosorbent assay (ELISA) kit (Cusabio, Wuhan, China).

### Immunohistochemical Analysis

Paraffin-embedded colon tissues (4 μm) were stained with occludin (1:200; Abcam, Hong Kong) or zonula occluden−1 (ZO-1, 1:100; Affinity Bioscience, United States) primary antibodies and incubated in a humidified chamber overnight at 4°C. The sections were then incubated with biotinylated goat anti−rabbit secondary antibodies (Boster Biological Technology, Wuhan, China) at room temperature for 20 min. Finally, color was developed in diaminobenzidine (DAB; ZSGB Biotechnology, Beijing, China) substrate solution. The average optical density (AOD) value of immunohistochemical intensity was analyzed by the Image J software (version 1.53a; National Institutes of Health, United States).

### Serum Lipopolysaccharide (LPS) and Liver LPS-Binding Protein (LBP) Quantitative Real Time-PCR Analysis

LPS, a component of the outer membrane of gram-negative bacteria, is an indicator of intestinal leakiness (Summa et al., 2013). The concentration of serum LPS was measured with commercially available limulus amebocyte lysate chromogenic kit (Dynamiker Biotechnology, Tianjin, China) according to the manufacturer’s protocol.

LBP is a type 1 acute phase protein that is constitutively produced by the liver and rapidly upregulated during acute phase responses. LBP binds LPS to facilitate immune responses in conjunction with cell-surface pattern recognition receptors and is used as an indicator of LPS exposure (Summa et al., 2013). Liver expression of LBP mRNA was assessed by quantitative real time -PCR. Primers sequence of LBP were as follows: F: 5′-TTACCGCCTGACTCCAACAT-3′, R: 5′-CAAGCCGGAAGACAGATTCG-3′). Quantification of LBP gene expression was performed using a ΔΔCt method with glyceraldehyde 3- phosphate dehydrogenase (GADPH) as an internal control.

### Statistical Analysis

All data were analyzed using SPSS 23.0 (IBM, Armok, United States.). A one-way analysis of variance (ANOVA) followed by Tukey’s *post hoc* test was used to identify statistical differences between groups. Data were shown as mean ± standard error of the mean. Statistical significance was defined as *p* < 0.05.

## Results

### Constant Light Exposure Aggravated Obesity and Visceral Adiposity in HFD Rats

**FIGURE 1:**
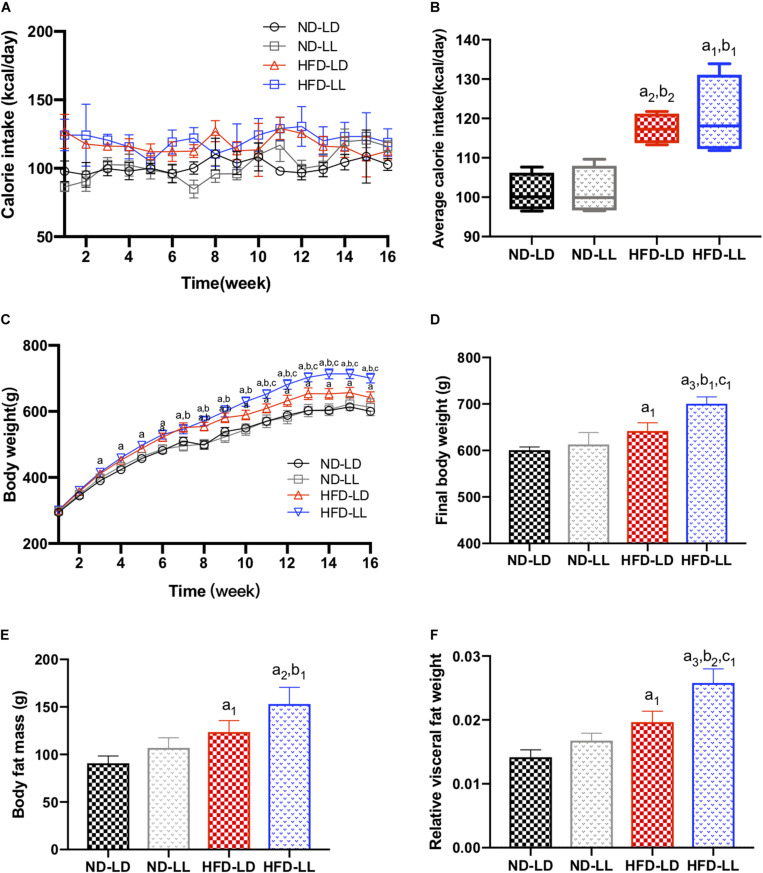
Constant light exposure aggravated obesity and visceral adiposity in HFD rats. **(A)** Calorie intakes, **(B)** average calorie intakes, **(C)** body weights, **(D)** final body weights, **(E)** body fat mass, and **(F)** relative visceral fat weight. Data were expressed as Mean ± SEM, *n* = 7 per group, ^a1^*p* < 0.05, ^a2^*p* < 0.01, ^a3^*p* < 0.001, vs. ND-LD group; ^b1^*p* < 0.05, ^b2^*p* < 0.01, vs. ND-LL group; ^c1^*p* < 0.05, vs. HFD-LD group.

There were no significant differences in food intakes, body weights, body fat mass, and relative visceral fat weights between ND-LD and ND-LL group (Figure 1). In HFD rats, body weights in HFD-LL group were significantly higher than those in HFD-LD group from the 11th week of HFD feeding, and no difference in caloric intake was noted between HFD-LD and HFD-LL groups (Figures 1A–D). Compared with the HFD-LD group, body weight and visceral fat weights in the HFD-LL group were significantly higher after 16 weeks (Figures 1E,F).

### Effects of Constant Light Exposure on Glucose Homeostasis and Serum Lipid Profiles

**FIGURE 2:**
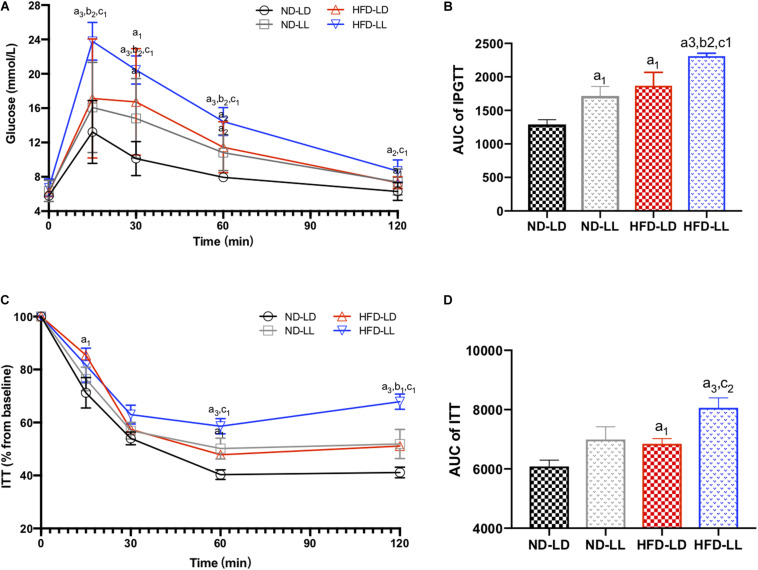
Effects of constant light exposure on glucose homeostasis. **(A)** IPGTT, **(B)** AUC of IPGTT, **(C)** ITT (% basal), and **(D)** AUC of ITT. Data were expressed as Mean ± SEM, *n* = 7 per group, ^a1^*p* < 0.05, ^a2^*p* < 0.01, ^a3^*p* < 0.001, vs. ND-LD group; ^b1^*p* < 0.05, ^b2^*p* < 0.01, vs. ND-LL group; ^c1^*p* < 0.05,^c2^*p* < 0.01 vs. HFD-LD group.

In normal chow fed rats, serum TC in the ND-LL group was significantly higher than that of the ND-LD group (Table 1). During the IPGTT, blood glucose levels at 30and 60 min in the ND-LL group were higher than those noted in the ND-LD group. The area under the curve (AUC) of the IPGTT in the ND-LL group was higher than that in the ND-LD group (Figures 2A,B). ITT suggested an increased tendency of AUC_ITT_ in the ND-LL group vs. the ND-LD group (*p* = 0.0844) (Figures 2C,D).

**TABLE 1:** Comparison of serum lipids, AST/ALT and serum IL-6, TNF-α among groups at the end of the experiment.

**Group**	**ND-LD**	**ND-LL**	**HFD-LD**	**HFD-LL**
n	7	7	7	7
TG (mmol/L)	0.740.10	0.800.27	0.860.07	0.820.17
TC (mmol/L)	1.410.06	1.710.08^a^	1.710.09^a^	2.050.12^a,b,c^
HDL-C (mmol/L)	0.870.07	0.860.05	0.650.03^a,b^	0.630.04^a,b^
LDL-C (mmol/L)	0.480.05	0.540.03	0.740.05^a,b^	0.930.07^a,b,c^
AST(U/L)	92.566.95	106.6310.08	129.8921.61	209.7433.07^a,b^
ALT(U/L)	36.243.09	33.312.95	41.119.03	45.598.33
AST/ALT	2.560.16	3.240.19^a^	3.470.30^a^	4.790.22^a,b,c^
IL-6 (pg/mL)	26.821.65	40.262.75^a^	46.262.21^a,b^	70.012.10^a,b,c^
TNF-α (pg/mL)	8.380.51	8.860.15	11.840.61^a,b^	14.920.59^a,b,c^

*Results were expressed as Mean ± SEM. ^*a*^p < 0.05, compared with ND-LD group. ^*b*^p < 0.01, compared with ND-LL group. ^*c*^p < 0.01, compared with HFD-LD group. TG, triglyceride; TC, total cholesterol; HDL-C, high-density lipoprotein cholesterol; LDL-C, low-density lipoprotein cholesterol; AST, aspartate aminotransferase; ALT, alanine aminotransferase; IL-6, interleukin-6; TNF-α, tumor necrosis factor-alpha.*

In HFD rats, serum TC and LDL-C in the HFD-LL group was significantly higher than those in the HFD-LD group (Table 1). During IPGTT, the HFD-LL group displayed increased levels of blood glucose starting at 15 min post-glucose injection vs. the HFD-LD group (Figure 2A). ITT demonstrated greater insulin resistance in the HFD-LL group vs. the HFD-LD group (Figure 2D).

### Constant Light Exposure Promotes the Progress of NAFLD in HFD Rats

**FIGURE 3:**
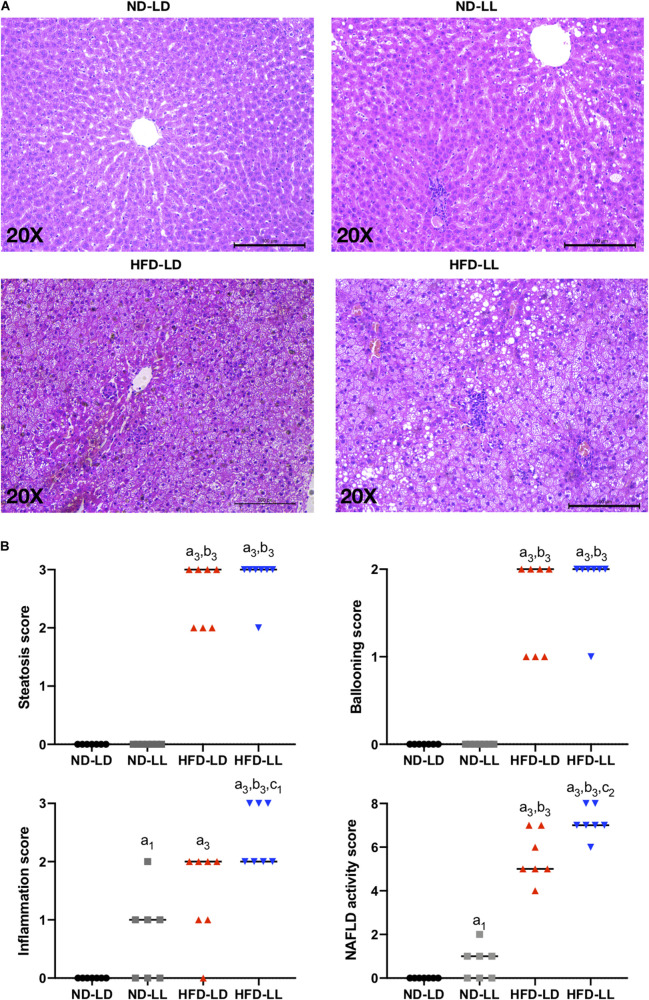
Constant light exposure aggravated the progression of NAFLD/NASH. **(A)** Representative pictures of H&E staining (200 × magnifications) in liver tissue. **(B)** NAFLD activity score. Data were expressed as Median, ^a1^*p* < 0.05, ^a3^*p* < 0.001, vs. ND-LD group; ^b3^*p* < 0.001, vs. ND-LL group; ^c1^*p* < 0.05, ^c2^*p* < 0.01, vs. HFD-LD group.

Compared with the ND-LD group, the ND-LL group had increased serum IL-6 concentration and AST/ALT ratio (Table 1). Liver histopathological examination showed more inflammatory cell infiltration in the ND-LL group as well (Figures 3A,B).

In HFD rats, hepatic steatosis and inflammatory infiltrates were significantly elevated in the HFD-LL group (Figure 3A). Also, the HFD-LL group had higher NAS and inflammation scores than the HFD-LD group (*p* < 0.05, Figure 3B). Serum TNF-α, IL-6, and AST/ALT ratio were also higher in the HFD-LL group (Table 1).

### Constant Light Exposure Altered Intestinal Microbial Communities

**FIGURE 4:**
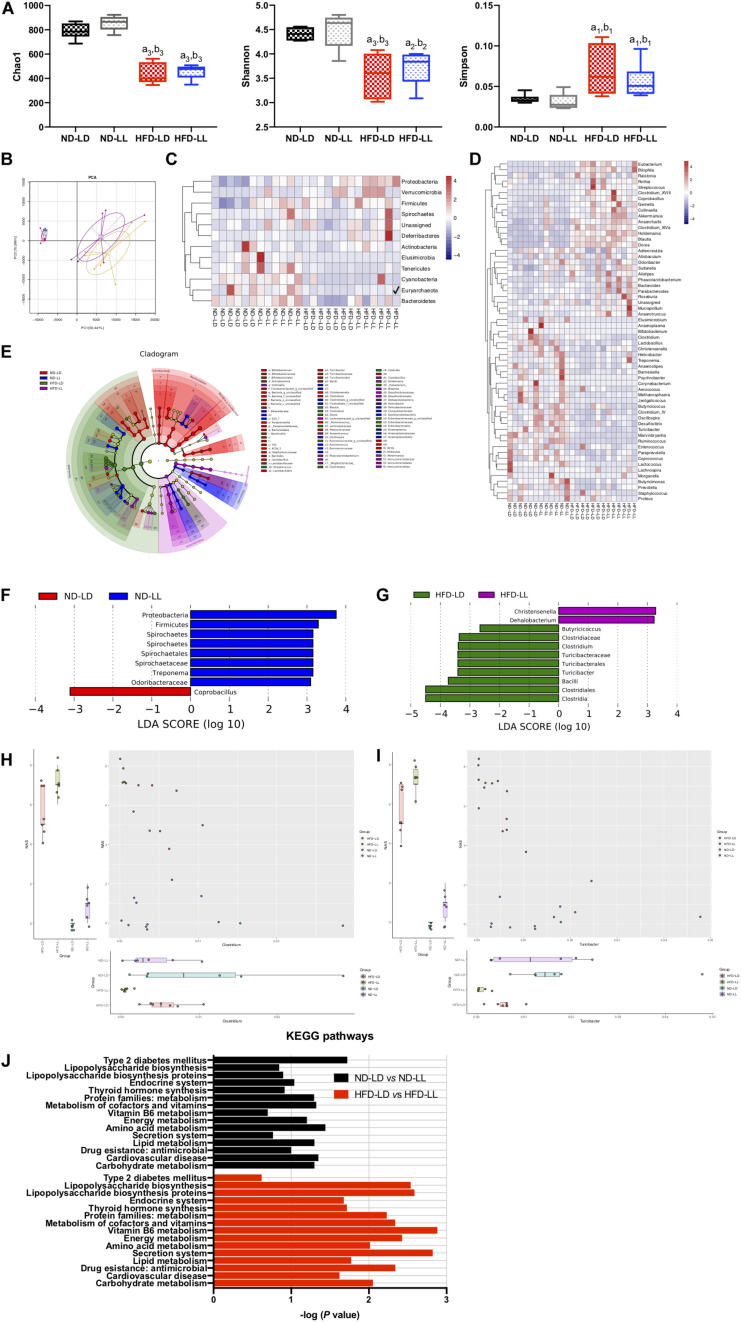
Constant light exposure altered colon microbiota. **(A)** Alpha diversity of colon microbiota estimated by Chao 1, Shannon and Simpson indexes, **(B)** principle component analysis (PCA), **(C,D)** heatmap of colon microbiota at phylum **(C)** and genus **(D)** level, **(E)** cladogram, **(F,G)** scores of taxonomic biomarkers identified by linear discriminant analysis (LDA) using LEfSe in normal chow-fed rats **(F)** and HFD-fed rats **(G)**, LDA value > 2.5 were showed in the figure, **(H,I)** correlation between NAS and abundance of *Clostridium* **(H)**, *Turicibacter* **(I)** revealed by generalized estimated equation (GEE) analysis, **(J)** KEGG pathways changed by constant light exposure. ^a1^*p* < 0.05, ^a2^*p* < 0.01, ^a3^*p* < 0.001, vs. ND-LD group; ^b1^*p* < 0.05, ^b2^*p* < 0.01, ^b3^*p* < 0.001, vs. ND-LL group.

There were no significant differences in alpha diversity and beta diversity between the ND-LD and ND-LL groups (Figures 4A,B). LEfSe analysis showed the relative higher abundance of *Protobacteria*, *Firmicutes, Spirochaetes* phylum, *Spirochaetes* class, *Spirochaetales* order, *Spirochaetaceae*, and *Odoribacteraceae* families, and *Treponema* genus in the ND-LL group compared to the ND-LD group (Figure 4F).

Compared with the ND-LD group, HFD-LD rats had decreased alpha diversity, increased abundance of *Firmicutes*, *Proteobacteria*, and *Verrucomicrobia* and decreased abundance of *Bacteroides* (Figures 4C–E).

The alpha diversity did not reveal significant differences between the HFD-LL and HFD-LD groups. The PCA represented structural change by the second principal component (PC2) with no statistic differences in PC1 (Figures 4A,B). The LEfSe analysis demonstrated constant light exposure restrained the growth of genus *Butyricicoccus*, *Clostridium*, *Turicibacter*, and class *Bacilli* in HFD fed rats. Compared with the HFD-LD group, the amounts of *Christensenella* and *Dehalobacterium* were higher in the HFD-LL group (Figure 4G). Consistent with the notion that constant light exposure causes increased severity of NASH, GEE analysis demonstrated that NAS was inversely correlated with genus *Turicibacter* (*p* < 0.01) and genus *Clostridium* (*p* < 0.05) (Figures 4H,I).

Pathways related to type 2 diabetes mellitus were elevated in the ND-LL group vs. those of the ND-LD group. Compared with the HFD-LD group, LPS biosynthesis phagosome, LPS biosynthesis proteins, Vitamin B6 metabolism, and antimicrobial drug resistance were significantly upregulated in the HFD-LL group (Figure 4J).

### Effects of Constant Light Exposure on SCFAs/MCFAs Levels of Colon Content

**FIGURE 5:**
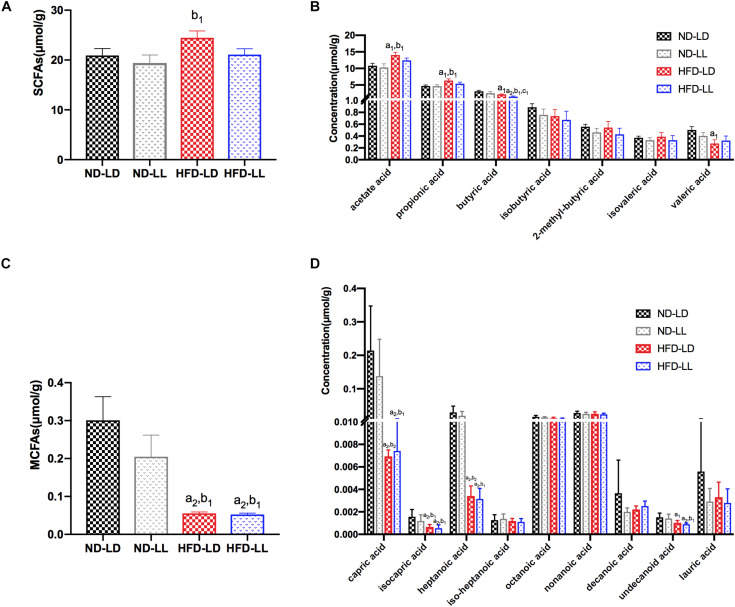
Effects of constant light exposure on short chain fatty acids (SCFAs) and medium chain fatty acids (MCFAs) of colon contents. **(A)** Total amount of SCFAs, **(B)** SCFAs species, **(C)** total amount of MCFAs, **(D)** MCFAs species. Data were expressed as Mean ± SEM, ^a1^*p* < 0.05, ^a2^*p* < 0.01, vs. ND-LD group; ^b1^*p* < 0.05, vs. ND-LL group; ^c1^*p* < 0.05, vs. HFD-LD group.

There were no significant differences in SCFAs and MCFAs levels of colon contents between ND-LD and ND-LL groups. Compared with ND-LD group, an increase in acetate acid, propionate acid, and a decrease in butyrate acid, valerate acid, and MCFAs in colon contents were detected in the HFD-LD group. Among HFD groups, reduced butyrate acid was observed in the HFD-LL rats (Figures 5A–D).

### Constant Light Exposure Caused Gut Barrier Dysfunction

**FIGURE 6:**
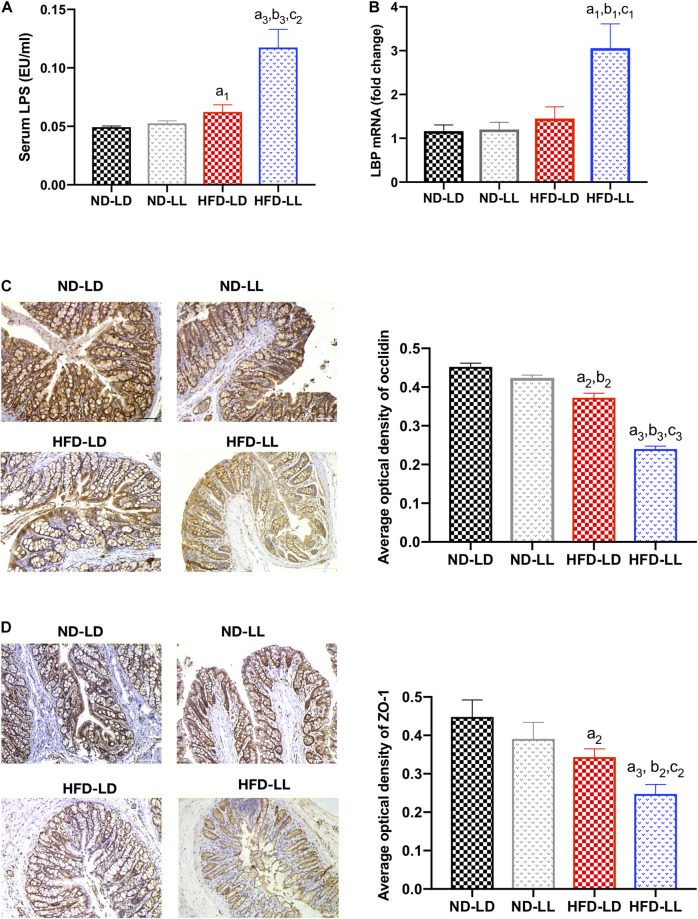
Constant light exposure impaired gut barrier function and gut-liver axis in HFD rats. **(A)** Serum LPS concentration, **(B)** hepatic LBP mRNA expression by RT-PCR, **(C)** expression of occludin in colon by IHC (200×), **(D)** expression of ZO-1 in colon by IHC (200×). Data were expressed as Mean ± SEM, ^a1^*p* < 0.05, ^a2^*p* < 0.01, ^a3^*p* < 0.001, vs. ND-LD group; ^b1^*p* < 0.05, ^b2^*p* < 0.01, ^b3^*p* < 0.001, vs. ND-LL group; ^c1^*p* < 0.05,^c2^*p* < 0.01, ^c3^*p* < 0.001 vs. HFD-LD group.

Gut barrier dysfunction plays an important role in the progression of NASH (Xue et al., 2017). Imbalances of gut microbiota and decreased SCFAs (e.g., butyrate acid) are associated with gut barrier dysfunction (Hamer et al., 2008; Lee et al., 2019). Compared to the control (ND-LD) group, HFD groups had lower expression of occludin and ZO-1 in proximal colon, and significantly higher circulating LPS levels and liver LBP mRNA expression. In HFD rats, constant light exposure caused a further increase in serum LPS and liver LBP mRNA expression, with further decrement of occludin and ZO-1 expression (all *p* < 0.05) (Figures 6A–D).

## Discussion

With the widespread prevalence of electric lights, particularly at night, light pollution is rising by approximately 6% per year worldwide (Holker et al., 2010). Light pollution is expected to rise dramatically in the next several decades through more urban development, such as street lighting, vehicles lighting, and security lighting. However, consequences of light pollution remain largely unknown. In this report, we demonstrated that constant light exposure predisposed HFD rats, a widely used animal model of obesity and NAFLD, to increased obesity and NAFLD/NASH progression. This suggests that light pollution is a novel risk factor for NAFLD/NASH progression. In normal chow fed rats, ND-LL rats showed increased levels of TC and blood glucose, and higher levels of IL-6, also suggesting deleterious effects of constant light exposure on metabolism.

Gut dysbiosis is associated with the development of NAFLD (Tripathi et al., 2018). Studies suggest that the development of NAFLD is associated with marked changes of fecal microbiota composition (Aron-Wisnewsky et al., 2013; Wang et al., 2018). In addition, results of both animal and human studies suggest that targeting intestinal microbiota may have protective effects on the development of NAFLD (Tripathi et al., 2018). However, changes in gut microbiota were not entirely consistent among studies (Kolodziejczyk et al., 2019). For example, among NAFLD individuals, an increase in *Proteobacteria* and *Bacteroidetes* has been reported (Boursier et al., 2016; Shen et al., 2017), while a reduction in *Proteobacteria* and *Bacteroidetes* was also observed (Shin et al., 2017; Koopman et al., 2019). Different ethnicities and living environments may help explain these discrepancies. Our experiment showed an increase of *Firmicutes*, *Proteobacteria*, *Verrucomicrobia*, and a decrease of *Bacteroides* in the HFD-LD vs. ND-LD group.

Evidences indicate that circadian clock has a profound impact on gut microbiota (Liang et al., 2015; Voigt et al., 2016). Circadian disruption by constant darkness or *Clock*^Δ^ ^19^ mutation changes the composition and diurnal oscillation of gut microbiome in normal chow fed mice (Voigt et al., 2014, 2016; Wu et al., 2018). In normal chow fed rats, we found that constant light exposure increased *Proteobacteria* and *Firmicutes*. It has been reported that *Proteobacteria* ferment to produce alcohol, and higher concentrations of endogenous alcohols are thought to contribute to liver injury and inflammation (Leung et al., 2016). Thus, the increased abundance of *Proteobacteria* may explain the increased AST/ALT and liver inflammatory cells infiltration in the ND-LL group.

Very few experiments have been performed to study the effects of constant light exposure on gut microbiome in HFD-fed animals. The present study was the first to note increased *Christensenella* and *Dehalobacterium*, and a decrease in genus *Butyricicoccus*, *Clostridium*, *Turicibacter*, and class *Bacilli* in HFD rats exposed to constant light. *Clostridium* has been associated with improved lipid regulation, reduced risk of hyperlipidemia, intestinal permeability, inhibition of harmful pathogens, and normalization of lipid metabolism (Ling et al., 2016). In NAFLD patients, a negative correlation between severity of NASH and abundance of *Clostridium* has been reported (Pataky et al., 2016; Zhou et al., 2019). The present animal study also demonstrated a negative correlation between NAS and abundance of genus *Clostridium*.

Another interesting discovery of our study was that constant light exposure caused decreased butyrate acid levels in colon content of HFD rats. The impact of gut microbiota on NAFLD is considered to be mediated by its metabolic products, such as SCFAs. Among SCFAs, butyrate has been reported to upregulate the expression of tight junction proteins, and consequently can enhance the gut barrier function (Lu et al., 2016). Butyrate supplementation to animals has been demonstrated to reduce hepatic fat accumulation and hepatic inflammation (Canfora et al., 2019). The decreased butyrate level in the HFD-LL group is consistent with the reduction in *Clostridium* genus, as the *Clostridium* genus has butyrate-producing effects (Vital et al., 2014; Ganesan et al., 2018). Targeting gut microbiota therapy has protective effects on the development of NAFLD (Tripathi et al., 2018). Light pollution is sometimes inevitable, e.g., shift workers, medical staff, et cetera. It may be possible that supplementation of some bacteria (e.g., *Clostridium* genus) or butyrate acid can be used as an intervention therapy for early-stage NASH caused by light pollution.

NAFLD is associated with increased gut permeability and impaired gut-liver axis (Jiang et al., 2015; Szabo, 2015). Gut hyperpermeability permits the translocation of proinflammatory bacterial products (most prominently, LPS) from the lumen of the gut into systemic circulation and promotes the progression of NASH (Okubo et al., 2016). We found that HFD-LL rats had decreased colon expression of tight junction proteins ZO-1 and occludin, and increased serum LPS level and liver LBP mRNA expression vs. the HFD-LD group, suggesting increased gut barrier dysfunction and impaired gut-liver axis exists in HFD rats exposed to constant light.

Taken together, the results presented herein demonstrate that constant light exposure alters gut microbiota and its metabolic products, impairs gut barrier function and gut-liver axis, and promotes the progression of NAFLD/NASH in HFD rats.

## Limitations

This study was the first to explore the impact of constant light exposure on NAFLD. However, there are some limitations to note. First, the light intensity was only set at 200 lux. Different results may occur when light intensity changes. Second, the illumination wavelength in our study was 400∼560 nm. It has been reported that circadian responses have different spectral sensitivity, peaking at wavelengths between 450 and 490 nm (Dominoni et al., 2016). This suggests that illumination with different wavelengths may have varying effects. Our experiment used a circadian clock-disrupting light source, though specifying which wavelength has the most impact cannot be determined. Last, we carried out the experiment only in male SD rats, though gender is thought to be an important factor in metabolic syndrome and its outcomes. There are gender differences in prevalence, risk factors, and mechanisms of NAFLD. It is still unknown the effects of constant light exposure on female subjects. Moreover, the number of animals was small.

## Conclusion

Constant light exposure changes gut microbiota and its metabolic products, impairs gut barrier function and gut-liver axis, and promotes the progression of NAFLD/NASH in HFD rats.

## Data Availability Statement

The datasets generated for this study can be found in the NCBI Sequence Read Archive under BioProject PRJNA597851 (https://www.ncbi.nlm.nih.gov/bioproject/PRJNA597851).

## Ethics Statement

The experiments were conducted according to the Animal Use and Care Committee of Central South University and were conducted according to the regulations set by Central South University (No. 2018sydw184).

## Author Contributions

LW and DZ conceived and designed the study. LW, FY, LX, SW, YS, and XX performed the experiments. LW, FY, JL, LX, SW, and YS analyzed the data. SM and GS analyzed the concentration of microbiota metabolites. LW and FY drafted the manuscript. DZ and RR revised the manuscript. All authors have read, commented on, and approved the manuscript for publication.

## Conflict of Interest

The authors declare that the research was conducted in the absence of any commercial or financial relationships that could be construed as a potential conflict of interest.

## Abbreviations

ALT: alanine aminotransferase
AST: aspartate aminotransferase
AUC: area under the curve
GEE: generalized estimated equation
HDL-C: high-density lipoprotein cholesterol
HFD: high fat diet
IHC: immunohistochemistry
IL-6: interleukin-6
IPGTT: intraperitoneal glucose tolerance test
ITT: insulin tolerance test
KEGG: Kyoto Encyclopedia of Genes and Genomes
LBP: LPS-binding protein
LDA: linear discriminant analysis
LDL-C: low-density lipoprotein cholesterol
LEfSe: LDA effect size
LPS: lipopolysaccharide
MCFAs: medium chain fatty acids
NAFLD: non-alcoholic fatty liver disease
NAS: NAFLD activity score
NASH: non-alcoholic steatohepatitis
ND: normal chow diet
OTUs: operational taxonomic units
PCA: principal component analysis
PICRUSt: phylogenetic investigation of communities by reconstruction of unobserved states
RT-PCR: reverse transcription polymerase chain reaction
SCFAs: short chain fatty acids
SCN: suprachiasmatic nuclei
TC: total cholesterol
TG: triglyceride
TNF-α: tumor necrosis factor-alpha
ZO-1: zonula occluden − 1.
